# An Updated Classification System and Review of the Lipooligosaccharide Biosynthesis Gene Locus in *Campylobacter jejuni*

**DOI:** 10.3389/fmicb.2020.00677

**Published:** 2020-05-19

**Authors:** Amber Hameed, Alexandra Woodacre, Lee R. Machado, Gemma L. Marsden

**Affiliations:** ^1^Division of Life Sciences, University of Northampton, Northampton, United Kingdom; ^2^Healthcare Infection Society, London, United Kingdom

**Keywords:** lipooligosaccharide, *Campylobacter jejuni*, ganglioside mimics, Guillain–Barré syndrome, Miller Fisher syndrome

## Abstract

Lipooligosaccharide (LOS) is an integral component of the *Campylobacter* cell membrane with a structure of core oligosaccharides forming inner and outer core regions and a lipid A moiety. The gene content of the LOS core biosynthesis cluster exhibits extensive sequence variation, which leads to the production of variable cell surface LOS structures in *Campylobacter*. Some LOS outer core molecules in *Campylobacter jejuni* are molecular mimics of host structures (such as neuronal gangliosides) and are thought to trigger neuronal disorders (particularly Guillain–Barré syndrome and Miller Fisher syndrome) in humans. The extensive genetic variation in the LOS biosynthesis gene cluster, a majority of which occurs in the LOS outer core biosynthesis gene content present between *lgtF* and *waaV*, has led to the development of a classification system with 23 classes (A–W) and four groups (1–4) for the *C. jejuni* LOS region. This review presents an updated and simplified classification system for LOS typing alongside an overview of the frequency of *C. jejuni* LOS biosynthesis genotypes and structures in various *C. jejuni* populations.

## Introduction

*Campylobacter* is a foodborne enteropathogen, which causes an acute, self-limiting gastroenteritis in humans with various non-specific symptoms including watery or bloody diarrhea, abdominal pain, headache, fever, chills, and dysentery (van Spreeuwel et al., 1985; Black et al., 1988; Perkins and Newstead, 1994). The annual estimated number for *Campylobacter* infection is 96 million worldwide (Havelaar et al., 2015). *Campylobacter* infection occurs in adults and children in developing countries and can also lead to death in young children (Janssen et al., 2008). In some cases, there can also be long-term, postinfection consequences of *Campylobacter* infection such as the neuronal disorders Guillain–Barré syndrome (GBS) and Miller Fisher syndrome (MFS), Reiter’s arthritis, and irritable bowel syndrome (IBS; Endtz et al., 2000; McCarthy and Giesecke, 2001). Cohort studies on confirmed *Campylobacter* cases estimate that GBS develops following infection in anywhere from 21 to 172 per 100,000 *Campylobacter* cases and that this rate is approximately 100-fold higher than in the general population (McCarthy and Giesecke, 2001; Tam et al., 2006; Scallan Walter et al., 2019). Case–control studies on patients diagnosed with GBS repeatedly show a significant association with *Campylobacter*, with an average rate of infection of 35.4% compared with 4.4% in controls (Poropatich et al., 2010). A recent systematic review of all factors contributing to the development of GBS concluded that *Campylobacter* infection was the most common trigger of the disease with a substantial evidence base (Wachira et al., 2019).

*Campylobacter* species, similar to *Neisseria* and *Haemophilus*, lack lipopolysaccharide (LPS) in the outer-cell membrane and instead possess lipooligosaccharide (LOS), which comprises of lipid A and core structures (Mandrell et al., 1992; Moran, 1997; Duncan et al., 2009). In comparison to LPS, LOS are low-molecular weight biological molecules lacking *O* chains (Moran, 1997). Other *Campylobacter* cell*-*surface structures include capsular polysaccharides (CPS), *O-*linked glycosylated flagellum, and *N-*linked glycoproteins. LOS, CPS, and *O*-linked glycans (mainly flagellar glycans) are variable among different strains, while *N-*linked glycoproteins remain conserved (Szymanski et al., 2003; Karlyshev et al., 2005; Day et al., 2012). The glycome that comprises these four types of carbohydrate containing conjugate molecules is synthesized by more than 8% of the genome in *Campylobacter jejuni* 11168 (Parkhill et al., 2000; Gundogdu et al., 2007).

No commercial vaccine has been developed for *Campylobacter* to date, and this is largely due to the versatile and diverse nature of *Campylobacter* physiology and genomics (Riddle and Guerry, 2016). Subunit vaccines formed with flagellum-secreted proteins (*C. jejuni* 81-176 FlaC, *C. jejuni* 81-176 FspA1, and *C. jejuni* CG8486 FspA2) and recombinant protein (ACE 393) have been experimentally tested in mice and healthy volunteers, respectively, but no promising candidates for human vaccines have been identified (Baqar et al., 2008; Poly et al., 2019). Glycoconjugate vaccines such as *C. jejuni* 81–176 conjugated CPS vaccine (CRM-197) has been tested in monkeys but remained unsuccessful, as it did not provide adequate immunity (Monteiro et al., 2009). A conjugated LOS vaccine has not been investigated yet for *C. jejuni*; however, LOS of two *C. jejuni* strains BH-01-0142 and CG8421 may be considered and utilized for vaccine development (Poly et al., 2019). LOS functions as a virulence determinant, immune modulator, and essential survival element, which make it a potential glycoconjugate vaccine candidate. However, prevalence of diverse LOS genotypes (due to variation within the gene content of LOS biosynthesis gene cluster) and presence of phase variation (a phenomenon where gene on/off switching varies the cell-surface LOS structures and functions) within the LOS biosynthesis genes are the two main features that make LOS less desirable as a vaccine candidate (Guerry et al., 2002; Gilbert et al., 2002; Parker et al., 2005, 2008). Furthermore, it is unclear how LOS genotypes are reflected in overall LOS biosynthetic structures. The prevalence of LOS genotypes vary from geographic region to region, while phase variation varies from strain to strain, and both types of LOS locus variation need to be investigated for vaccine design. The most prevalent LOS genotypes circulating regionally must be taken into account for maximal efficiency of LOS-conjugated vaccine, and therefore, the frequency of *C. jejuni* LOS genotypes in different countries including United States, United Kingdom, Netherlands, France, Belgium, Finland, Sweden, Japan, and Bangladesh has been investigated previously (Godschalk et al., 2004; Parker et al., 2005; Quiñones et al., 2007; Habib et al., 2009; Ellström et al., 2013, 2014, 2016; Islam et al., 2014, 2018; Ohishi et al., 2017; Elhadidy et al., 2018; Thépault et al., 2018). In this review, data from the literature relevant to the distribution of *C. jejuni* LOS locus genotypes in various geographical areas of the world will be analyzed to present an up-to-date picture of *C. jejuni* LOS genotype predominance, which may be important in vaccine design. This review also presents an updated and simplified classification system for the LOS biosynthesis locus to aid fellow researchers investigating increasingly complex levels of LOS variation and the role it plays in *Campylobacter* infection.

## *Campylobacter* Los as a Virulence Determinant

**FIGURE 1:**
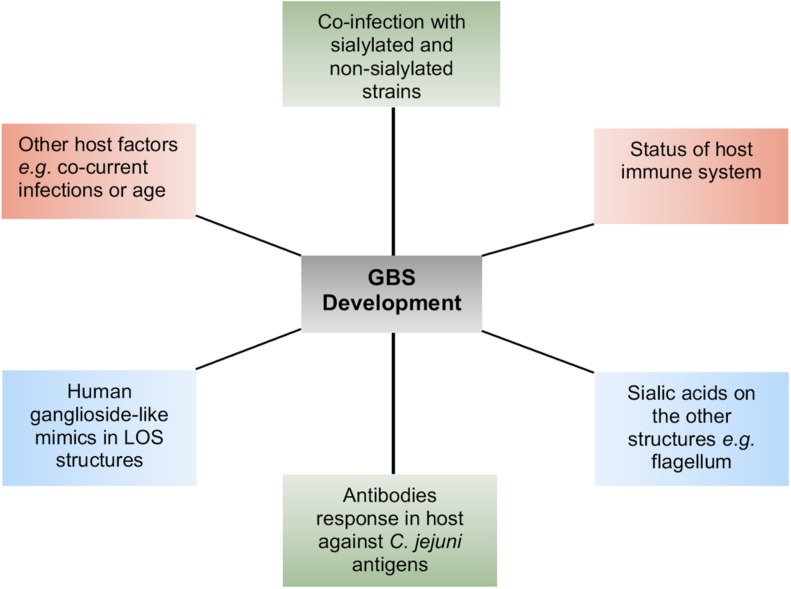
A summary of the different host–pathogen factors that contribute to the complexity of Guillain–Barré syndrome (GBS) development.

LOS in *Campylobacter* does not only maintain the integrity of the cell membrane structure but also acts as a barrier for those molecules, which are transported through the cell membrane (Karlyshev et al., 2005). Deletion of LOS core in *C. jejuni* 11168 does not seem essential for viability (Marsden et al., 2009), but truncation of LOS can be lethal in *C. jejuni* strains other than 11168 (Phongsisay et al., 2007). For example, antibiotic permeability into the cell increases due to alteration in LOS structures, possibly because LOS structural changes decrease the cell membrane hydrophobicity. This is the reason that mutants of *C. jejuni* LOS genes are highly susceptible to some antibiotics, specifically to erythromycin (Kanipes et al., 2004; Jeon et al., 2009; Marsden et al., 2009). In addition to providing a barrier to antibiotics, LOS also confers resistance to *Campylobacter* cells against human serum proteins including α-defensins, cathelicidins, and bactericidal-/permeability-increasing proteins (Marsden et al., 2009; Keo et al., 2011). DNA uptake into a bacterial cell is an outer cell membrane-dependent process. Therefore, LOS modification in the outer cell membrane may also affect *Campylobacter*’s ability to uptake foreign DNA or its characteristic of natural transformation (Jeon et al., 2009; Marsden et al., 2009). Mutants of *Campylobacter* LOS genes, in comparison to their respective wild-type (WT) strains, have showed reduced adherence and invasion into host intestinal epithelial cells (Fry et al., 2000; Kanipes et al., 2004; Javed et al., 2012), which might be due to reduced interaction between host cell receptors and altered LOS structures. A caveat to these studies is that deletion of LOS biosynthesis genes or drastic changes in LOS structure may have a general destabilizing effect on the LOS. A mutant of *C. jejuni* 11168, lacking the core oligosaccharides in its LOS structures, was unable to invade Caco-2 cells, indicating the importance of LOS in *Campylobacter* invasion into host cells (Marsden et al., 2009). *C. jejuni* strains with sialylated LOS showed higher potential of adhesion, invasion, and translocation than those with non-sialylated LOS (Louwen et al., 2012). Two sialic acid biosynthesis genes (*cgtB* and *wlaN*) were found commonly present in highly invasive *C. jejuni* strains (Müller et al., 2007), and mutation of a sialic acid biosynthesis gene, *cst-II*, in a *C. jejuni* strain caused reduction in its invasion into epithelial cells (Louwen et al., 2008), supporting the role of LOS sialylation or sialic acid biosynthesis genes in *C. jejuni* invasion. However, *C. jejuni* mutants of other LOS biosynthesis genes (*waaC* and *cj1136*) also showed significant reduction in invasion into intestinal epithelial cells (Kanipes et al., 2008; Javed et al., 2012). Furthermore, only 23% of *C. jejuni* isolates from blood-borne infection or truly invasive strains contained sialic acid biosynthesis genes (Ellström et al., 2014). These studies indicate that not only sialic acid biosynthesis genes but also the overall presentation of LOS structure play an important role in adherence and invasion of *C. jejuni* into host cells. Complete cell-surface LOS structures in *C. jejuni* are also important for the optimum colonization of chick ceca, and this is linked to the increased hydrophobicity and susceptibility to bile of LOS mutants (Iwata et al., 2013). Thus, LOS is an important virulence determinant in *C. jejuni*. It may also be the case that since *C. jejuni* LOS are densely present on the cell surfaces and they are readily available to stimulate and interact with human immune cells such as macrophages, LOS could also contribute to the binding of other cell types. For example, the *C. jejuni* LOS terminal *N*-acetyl galactosamine residues bind to the human macrophage galactose-type lectin receptors (van Sorge et al., 2009). LOS containing sialic acid residues have particular significance for human disease due to their increased ability to bind to immune cells and their similarity to neuronal structures. *C. jejuni* LOS sialic acid residues bind to Toll-like receptor 4 (TLR-4) and sialoadhesin receptors present on the human macrophage cell surfaces (Klaas et al., 2012; Heikema et al., 2013; Stephenson et al., 2013). *C. jejuni* LOS sialic acid residues are also ligands of sialic acid binding immunoglobulin-like lectins present in human monocytes and natural killer cells (Avril et al., 2006). The LOS structures with variable epitopes presented on different *C. jejuni* cell surfaces can also mimic human neuronal gangliosides. For this reason, antibodies produced against the LOS structural epitopes do not only bind to LOS structures but also to human neuronal gangliosides. The cross-reactivity of anti-LOS antibodies with human gangliosides leads to the development of neuronal disorders (GBS and MFS) in humans (Yuki, 1997; Nachamkin et al., 1998; Endtz et al., 2000; McCarthy and Giesecke, 2001; Wakerley and Yuki, 2015). This is evident by the development of pathological changes in peripheral nerves and weakness in the limbs as well as production of anti-GM1 antibodies in rabbits upon sensitization with *C. jejuni* LOS (Yuki et al., 2004). Furthermore, knockout mutants of *C. jejuni* sialic acid biosynthesis genes (*orf10* and *cst-II*) with truncated and non-sialylated LOS structures show reduced reactivity with GBS patient serum. In addition, administration of these mutated LOS structures into mice did not induce antiganglioside antibody responses (Godschalk et al., 2004). The allelic variation in the *cst-II* gene leads to the expression of either threonine (Thr) or asparagine (Asn) at position 51 of the sialyltransferase (Gilbert et al., 2002). This genetic polymorphism (and change in host-mimicking ganglioside epitopes) can further affect the development of autoimmune and clinical symptoms of GBS, supporting the role of LOS gene variations in GBS (Koga et al., 2005). In GBS, the cranial nerves extending from the brain to various areas of the head and neck are affected, which further develop difficulty in walking, muscle weakness, and muscle pain, while MFS, a variant of GBS, is characterized mainly by paralysis of eye muscles and problems with balance and coordination (Nachamkin et al., 1998). These postinfection complications are infrequent and typically appear in immune-compromised individuals, such as individuals with HIV infection (McCarthy and Giesecke, 2001; Janssen et al., 2008). They do not develop solely as a consequence of *Campylobacter* infection, and other bacterial or host-specific risk factors aid in stimulating the production of antiganglioside antibodies (Figure 1; Revez and Hänninen, 2012; Islam et al., 2014). This varies among *C. jejuni* strains and *Campylobacter*-infected individuals and contributes to a complex picture of GBS development and progression postinfection (Godschalk et al., 2007; Müller et al., 2007). Some *C. jejuni* strains do not produce ganglioside mimicking LOS structures at all despite the presence of sialic acid biosynthesis genes, and therefore, the presence of sialylated LOS biosynthesis genes do not always correspond with the *C. jejuni* potential for neural disease development (Houliston et al., 2011).

## The Los Biosynthesis Locus in *C. jejuni*

**FIGURE 2:**
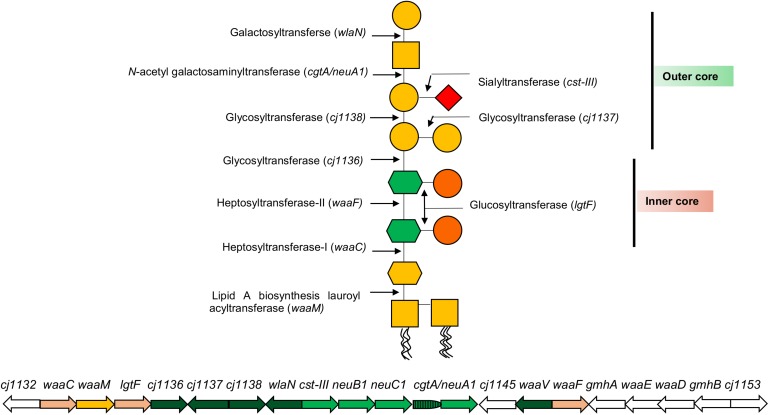
A representation of *C. jejuni* 11168 lipooligosaccharide (LOS) core biosynthesis gene cluster and its LOS structure. Each arrow represents an individual LOS core biosynthesis gene, and its direction indicates the direction of gene transcription. A LOS biosynthesis gene in yellow encodes an enzyme to catalyze the addition of a KDO molecule to lipid A. LOS biosynthesis genes in light pink encode enzymes for the synthesis of LOS inner core structure. LOS biosynthesis genes in green encode enzymes for the synthesis of LOS outer core structure, where LOS genes in light green (*neuB1*, *neuC1*, and *cgtA/neuA1*) synthesize sialic acid to incorporate into the outer core. LOS genes in white (*gmhA*, *waaE*, *waaD*, and *gmhB*) synthesize heptoses for inner core. Glycan structures were drawn according to the Symbol Nomenclature For Glycans (SNFG).

In proposing a new classification for the LOS, it is important to appreciate the complexity of the LOS biosynthesis loci that has informed rationale for current and historic classification systems. The LOS lipid A backbone in *C. jejuni* contains a 3-diamino-2,3-dideoxy-D-glucopyranose linked to 2-amino-2-deoxy-D-glucose (GlcN), whereas the *Campylobacter coli* lipid A backbone consists of two GlcN (Culebro et al., 2016). The lipid A backbone in most of the *Campylobacter* strains is linked to six acyl chains (two hydroxyl-linked and four amide-linked; Moran, 1997). The LOS core biosynthesis in *C. jejuni* is achieved at the genetic level by a cluster of LOS biosynthesis genes (Figure 2). Each LOS biosynthesis gene produces an individual enzyme involved in either monosaccharide biosynthesis or addition of a particular monosaccharide to the LOS structure (Karlyshev et al., 2005; Parker et al., 2005, 2008; Iwata et al., 2013). The inner core of *C. jejuni* LOS has two heptose and two glucose units (Klena et al., 1998; Gilbert et al., 2002; Kanipes et al., 2004, 2006). The heptosyltransferase-I (*waaC*) adds the first heptose (Hep-I) to 3-deoxy-D-manno-octulosonic acid (KDO). Heptosyltransferase-II (*waaF*) catalyzes the addition of a second heptose (Hep-II) to Hep-I (Klena et al., 1998; Kanipes et al., 2004, 2006). In *C. jejuni* (strain 11168), Hep-1 and Hep-II are synthesized and added to the inner core of LOS by the phosphoheptose isomerase (*gmhA*), a D-glycero-beta-D-manno-heptose-7-phosphate kinase (*waaE*), an ADP-L-glycero-D-manno-heptose-6-epimerase (*waaD*), and a dephosphatase (*gmhB*; Karlyshev et al., 2005; Iwata et al., 2013). Unlike the inner core, the outer core of LOS varies extensively among *C. jejuni* strains (Linton et al., 2000; Godschalk et al., 2004; Houliston et al., 2011). The outer core of *C. jejuni* 11168 is synthesized by glycosyltransferases [*cj1136* (*orf4*), *cj1137* (*orf14*), and *cj1138* (*orf15*)], *N*-acetyl galactosaminyl transferase [*cgtA*/*neuA1* (*orf5/10*)], sialyltransferase [*cst-III* (*orf7*)], and galactosyltransferase (*wlaN*) and is illustrated as an example in Figure 2 (Gilbert et al., 2000, 2002; Linton et al., 2000; Guerry et al., 2002; Karlyshev et al., 2005; Javed et al., 2012). In addition to core synthesis, a LOS biosynthesis gene (*waaM*) is located in the cluster that encodes an enzyme (lipid A biosynthesis lauroyl acyltransferase) to catalyze the addition of a KDO molecule to the backbone of lipid A (Karlyshev et al., 2005). *Campylobacter* LOS structures are synthesized in the cytoplasmic side of the inner cell membrane from where they are flipped to the periplasmic side of the inner cell membrane and, finally, are integrated into the outer cell membrane (Whitfield and Trent, 2014; Simpson et al., 2015). Based on similarities to LPS assembly machinery in *Escherichia coli*, it is predicted that *N*-linked glycosylation glycosyltransferase (*wlaM*/*pglG*) and flippase (*wlaB*/*pglK*) can respectively facilitate cytoplasm-to-periplasm LOS flipping and periplasm-to-outer cell membrane LOS translocation in *Campylobacter* (Fry et al., 1998).

## Variation in *C. jejuni* Los Biosynthesis Locus

The variation in the *C. jejuni* LOS biosynthesis gene region occurs either due to (i) mutations within the nucleotides of LOS biosynthesis gene sequences or (ii) the recombination between LOS biosynthesis gene/gene regions.

### Variation at the Nucleotide Level

Nucleotide level variations within the LOS biosynthesis genes can occur due to phase variation, where slip strand mispairing during the replication of homopolymeric tracts can lead to insertions or deletions of single bases (Gilbert et al., 2002). The LOS gene *wlaN* in *C. jejuni* 11168, *C. jejuni* 331, and *C. jejuni* 2500 containing a homopolymeric tract of 8G produces the fully transcribed and functional gene product β-1,3-galactosyltransferase (Linton et al., 2000; Müller et al., 2007; Semchenko et al., 2012). A variant in these strains containing a 9G homopolymeric tract in *wlaN* results in a frameshift mutation and premature translational termination with a non-functional gene product, which cannot add the terminal galactose in the LOS structure and consequently converts a GM1-like LOS epitope into a GM2 mimic (Linton et al., 2000; Semchenko et al., 2012). Site-directed mutagenesis of the homopolymeric tract in *C. jejuni* 11168 *wlaN* from 8G to 11G increases the rate of phase variation ∼10-fold in this gene, and in general, the rate of phase variation increases with longer tract lengths in multiple genes (Bayliss et al., 2012). Phase variation of *C. jejuni* 11168 *wlaN* was not observed *in vivo* during colonization of chickens aged 2–4 weeks, but an increase in tract length from 8G to 9G to switch off expression of *wlaN* was detected after the passage of *C. jejuni* 224 and 331 in 5-day-old chicks, with *C. jejuni* 11168-O remaining unchanged (Bayliss et al., 2012; Semchenko et al., 2012). In the same study, *C. jejuni* 331 switched off *wlaN* after coculture with the intestinal cell line CaCo-2 and *C. jejuni* 224 switched off expression of the LOS genes *Cj1144-45* after colonizing chicks, giving further evidence that strain- and host-specific factors can both influence phase variation of LOS genes (Semchenko et al., 2012). Phase variation in a number of other LOS biosynthesis genes has been observed at both the genotype and phenotype level in multiple strains including *cst-II*, *cgtA*, *cgtD*, *orf23*, and *orf25* (Guerry et al., 2002; Parker et al., 2005; Godschalk et al., 2006, Godschalk et al., 2007; Houliston et al., 2011; Wanford et al., 2018). Multiple combinations of phase variable genes can also lead to novel LOS structures. Different combinations of on and off phenotypes in *C. jejuni* (strain GC149) are encoded by the *cgtA* and *cgtD* outer core glycosyltransferases and result in structural molecular mimics of either GD3 (*cgtA* off), GT1a (*cgtA* on/*cgtD* off), or ganglio/Pk (*cgtA* on/*cgtD* on) gangliosides (Houliston et al., 2011). Phase variation of LOS genes can therefore lead to mixed populations of LOS gene variants and increase the diversity of LOS structural epitopes within a single strain of *Campylobacter* (Guerry et al., 2002).

Sequence variation may also occur due to single nucleotide mutations, which can inactivate the LOS biosynthesis genes without involving the phenomenon of phase variation. For example, deletion of an A-base at position 1234 in *lgtF* (a LOS biosynthesis gene) alters the catalytic activity of its encoded enzyme, glycosyltransferase, in four *C. jejuni* strains (ATCC 43432, ATCC 43446, OH4382, and OH4384). As a result, the produced glycosyltransferase does not have the potential to catalyze the addition of ß-1,2-glucose to heptose-II during the LOS synthesis. Similarly, the base substitution of the final base in *orf5/10* (*cgtA*/*neuA1*) in *C. jejuni* ATCC 43430 changes the amino acid (cysteine → tyrosine), which further leads to the production of a non-functional enzyme (Gilbert et al., 2002). The LOS gene, *cgtA*, with missing A-base at position 71 substitutes one amino acid in the *cgtA-*encoding enzyme, *N*-acetyl galactosaminyl transferase, which further leads to the inactivation of *N*-acetyl galactosaminyl transferase in *C. jejuni* OH4382 and OH4384 and truncates the LOS structure (Gilbert et al., 2002). Similarly, a five-base deletion from the *cst-III* gene of *C. jejuni* GB1 alters the number of amino acids (294→219) in sialyltransferase and eventually produces a non-sialylated LOS (Godschalk et al., 2007).

### Variation at Allele or Gene Level and LOS Locus Classes in *C. jejuni*

In recent years, an alphabetical system of class organization for LOS genes within the *C. jejuni* LOS biosynthesis locus has been developed based on 23 *C. jejuni* LOS classes (A through W), which have been previously described (Gilbert et al., 2002; Parker et al., 2008; Richards et al., 2013). An insertion or deletion of a LOS biosynthesis gene or gene regions into the LOS locus can give rise to a different class type (Parker et al., 2005, 2008). Alterations of portions of the LOS biosynthesis genes or different alleles can also establish a new class or subclass; for example, allele variation in *cgtA* and *wlaN* genes generates A and B subclasses including A1, A2, B1, and B2 (Parker et al., 2005). In addition, disruption in resident LOS biosynthesis genes can also form a new class; for instance, disruption in class E *orf26* establishes the LOS locus class P (Parker et al., 2005). The developed new locus type can be variable both in gene content and gene organization (Parker et al., 2005; Revez and Hänninen, 2012). *C. jejuni* acquires new genes in its LOS biosynthesis region by horizontal gene transfer. The horizontal transfer of LOS biosynthesis genes from *C. jejuni* O4 (GM1 strain) to *C. jejuni* 81116 (non-GM1 strain) changed it into a GM1-like LOS-producing strain (Phongsisay et al., 2006). Similarly, a *C. jejuni* GB11 strain possessing class C locus acquired a class A locus, identical to the LOS locus of *C. jejuni* ATCC 43446 while retaining the same sequence in the remainder of the genome (Gilbert et al., 2004).

Variation in LOS biosynthesis gene alleles causes alterations in the LOS structure. For instance, two *cst-II* gene alleles lead to the expression of either threonine (Thr) or asparagine (Asn) at position 51 of the translated enzyme. As a result, the enzyme retains either a monofunctional (Thr → 2,3-sialyltransferase activity) or a bifunctional (Asn → 2,3- and 2,8-sialyltransferase) activity and produces LOS with one and two sialic acids, respectively (Gilbert et al., 2002). Variation in LOS locus gene content can vary the carbohydrate content, linkages between the carbohydrate units, and core length in cell-surface LOS structures (Gilbert et al., 2002; Guerry et al., 2002). Variation in LOS locus gene content as well as in its gene organization varies the cell-surface LOS structurally and functionally. It is not always the case that LOS structures belonging to the same LOS locus type encode similar epitopes. *C. jejuni* 11168 and 520, both belong to class C, but *C. jejuni* 520 can produce a wider variety of human ganglioside mimics than *C. jejuni* 11168 (Semchenko et al., 2012). *C. jejuni* strains that contain a type A LOS locus frequently encode and express human ganglioside mimics on bacterial cell surfaces, which include GM1a, GM1b, GD1a, and GD1b (Nachamkin et al., 2002; Godschalk et al., 2004; Mortensen et al., 2009). For example, there is a GM1-like mimic in *C. jejuni* 11168 (class C), a GQ1b-like mimic in *C. jejuni* 81-176 (Class B), a Lewis type I-like mimic in *C. jejuni* RM1503 (class M), and a paragloboside/Pk-like antigens in *C. jejuni* RM1221 (class F; Godschalk et al., 2004; Mortensen et al., 2009; Houliston et al., 2011). *C. jejuni* GC149 (class R) contains sialic acid biosynthesis genes and may present ganglioside-like mimics (GT1a, GD3) as well as a hybrid form of ganglio and P-type antigens (Parker et al., 2008; Houliston et al., 2011). Other LOS classes such as D and E also possess human ganglioside-like LOS structures, but these are different to GM1, GD1, and GQ1b (Godschalk et al., 2004). Class P LOS have a lack of sialic acid and possess *N*-acetyl quinovosamine instead (Poly et al., 2008). The variable LOS structural epitopes presented by different *C. jejuni* LOS locus types are demonstrated in Table 1.

**TABLE 1:** Variable lipooligosaccharide (LOS) structures synthesized by different *C. jejuni* LOS locus types.

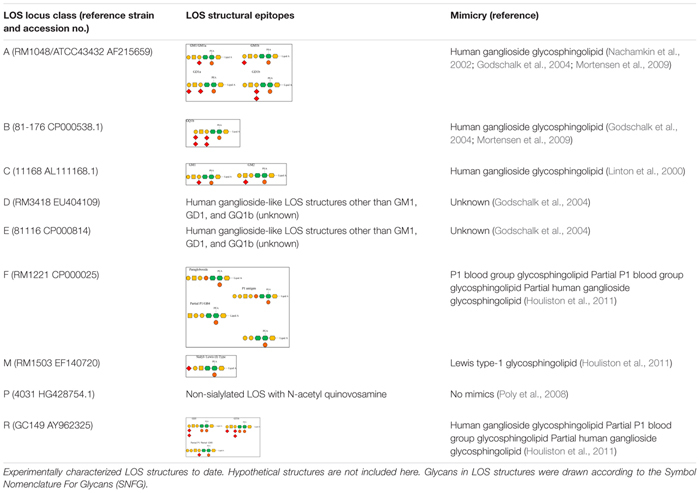

The expression of variable cell surface LOS structures and mimicry with human blood antigen glycosphingolipids or neuronal ganglioside glycosphingolipids as a consequence of gene variation in the LOS locus in *C. jejuni* is an important virulence factor and may have a direct link to the progression of specific neuronal disorders postinfection (Ang et al., 2002; Müller et al., 2007; Houliston et al., 2011; Semchenko et al., 2012). For example, *C. jejuni* strains with LOS locus class A and variable human ganglioside mimics (GM1a, GM1b, GD1a, and GD1b) trigger GBS in *Campylobacter*-infected patients (Nachamkin et al., 2002; Godschalk et al., 2004, 2007; Mortensen et al., 2009), whereas *C. jejuni* strains with LOS class B and corresponding GQ1b-like LOS structures are likely to develop MFS in *Campylobacter*-infected patients (Godschalk et al., 2007; Islam et al., 2014). Genetic diversity within the LOS locus plays an important role in the development of postinfection effects; however, this does not have any association with acute-phase symptoms such as diarrhea or abdominal pain (Poly et al., 2008; Mortensen et al., 2009; Ellström et al., 2013). This indicates that LOS sialylation is not required for human diarrheal disease and that both the sialylated and non-sialylated LOS can be used for vaccine design (Poly et al., 2008, 2019).

## Simplification of *C. jejuni* Los Locus Classification

**FIGURE 3:**
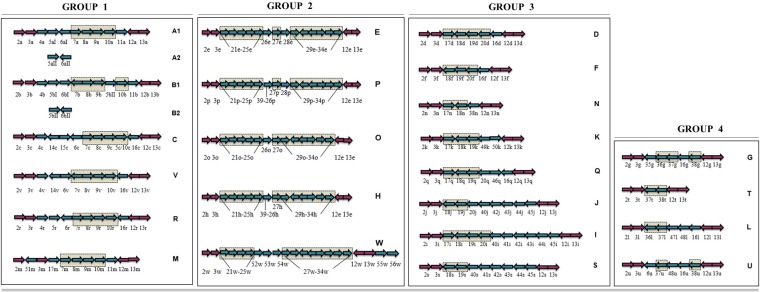
Simplified *C. jejuni* lipooligosaccharide (LOS) locus classification system. LOS classes are classified into the previously established four groups on the basis of sharing similar LOS biosynthesis gene content. Arrows in checked boxes: LOS biosynthesis gene content, shared between the classes within a LOS group; Blue arrows: Variable LOS biosynthesis genes, located between *lgtF* (*orf3*) and *waaV* (*orf12*); pink arrows: LOS biosynthesis genes, commonly present in all LOS classes. Genes are numbered according to the Parker et al. (2005) numbering system. Arrow direction represents the direction of gene transcription.

LOS classes A–H were initially described (Gilbert et al., 2002; Parker et al., 2005), and these known *C. jejuni* LOS classes were then primarily categorized into four groups and included LOS classes A, B, and C belonging to a group 1, LOS class E in group 2, LOS classes D and F in group 3, and LOS class G in group 4 (Karlyshev et al., 2005). Later, Parker et al. (2008) identified 11 more *C. jejuni* LOS classes including I–S. Subsequently, Richards et al. (2013) identified *C. jejuni* strains with novel LOS loci and established four more LOS classes including T, U, V, and W. The novel LOS loci identified in the latter two studies have never been assigned to the LOS groups. To better understand the prevalence of *C. jejuni* LOS groups and groups related to LOS classes, we propose a simplified LOS classification system (Figure 3) where various already known LOS classes have been assigned into the pre-established LOS groups (Karlyshev et al., 2005) on the basis of sharing similar LOS biosynthesis gene content. Group 1 includes all the LOS locus types (A, B, C, R, M, and V), which contain genes for sialic acid synthesis and translocation (*orf7*/*cst-II*/*cst-III*, *orf8*/*neuB1*, *orf9*/*neuC1*, and *orf10*/*neuA1*), whereas the other three groups have LOS loci with no sialic acid biosynthesis genes. Based on sequence similarity of LOS loci H, O, P, and W to locus E (*orf21–orf34*), these four classes are now assigned to group 2. Furthermore, K, Q, N, I, J, and S sharing *orf17*, *orf18*/*cgtH*, *orf19*/*cgtG*, and *orf20*/*cgtE* are assigned to LOS group 3, and L, G, T, and U sharing *orf36*, *orf37*, and *orf38* are assigned to LOS group 4 classes.

## Prevalence of *C. jejuni* Los Locus Classes and Groups

**FIGURE 4:**
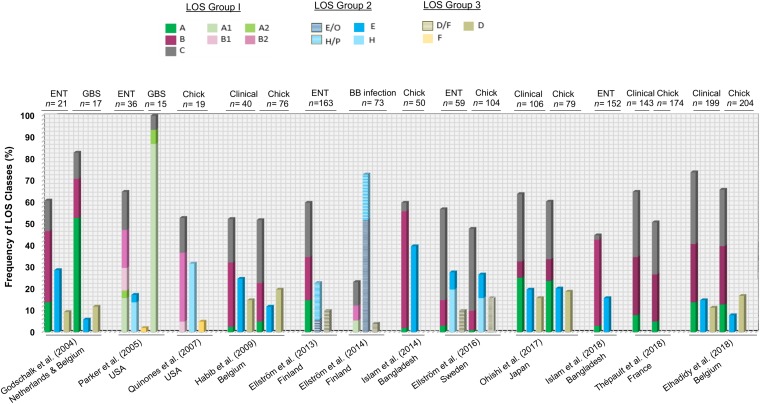
A comparison of previous findings of distribution of *C. jejuni* lipooligosaccharide (LOS) locus genotypes in various *C. jejuni* populations. ENT, *C. jejuni* isolates from enteritis patients; GBS, *C. jejuni* isolates from GBS patients; chick, *C. jejuni* isolates from chicken; BB infection, *C. jejuni* isolates from patients with blood-borne infections.

A large number of available *C. jejuni* genomes with metadata have been deposited in recent years and have the potential to provide a full and comprehensive overview of the frequency of *C. jejuni* LOS genotypes in *C. jejuni* populations (from different isolation sources and various clonal complexes). However, much of these data remain unpublished and should be a focus of ongoing efforts. However, analysis of previously published studies examining the frequencies of *C. jejuni* LOS locus classes and groups present in enteritis, GBS, blood borne infection, and poultry-associated *C. jejuni* populations indicates that the hierarchy of LOS group (group 1 > group 2 > group 3 > group 4) is largely conserved among human- and poultry-derived *C. jejuni* isolates (Figure 4). This also indicates that the B and C LOS classes in clinical enteric disease and LOS class A in GBS-associated *C. jejuni* populations are found to be highly predominant. In comparison to the high prevalence of LOS class C (42%) in clinical isolates in Sweden (Ellström et al., 2016), a very small number of clinical strains (2%) in Bangladesh had association with LOS locus C (Islam et al., 2018), suggesting that *C. jejuni* LOS class distribution may vary geographically. Furthermore, when comparing the combined frequency of LOS ABC types in different populations of *C. jejuni* isolates (clinical, enteritis, and poultry), approximately 50–75% of strains in all *C. jejuni* populations belong to LOS classes A, B, or C. The only exception to these results was the data from Ellström et al. (2014) where *C. jejuni* were isolated from human blood-borne infections.

A definite correlation between *C. jejuni* LOS locus class prevalence and sequence type (ST) distribution has not been established yet due to the diverse population structure of *C. jejuni* (Habib et al., 2009; Islam et al., 2014). A few studies have shown concordance between specific STs and LOS classes. For example, LOS class B possessing GBS (50%) and enteritis (25%) *C. jejuni* isolates had ST-403 CC (Islam et al., 2009). Another study assigned class B diarrheal *C. jejuni* strains (3%) with ST-206 CC (Habib et al., 2009). LOS class C was associated with ST-21 CC in 14% of *C. jejuni* enteritis isolates (Revez and Hänninen, 2012), 11.2% of *C. jejuni* bacteremia isolates (Ellström et al., 2013), and 3.2% of diarrheal *C. jejuni* isolates (Habib et al., 2009). High frequency of LOS locus class C may be a contributor to the high predominance of clinical *C. jejuni* strains with ST-21 CC (Habib et al., 2009; Thépault et al., 2018). This ST-21 CC has also been found in LOS class A positive bacteremia (2.8%) and diarrheal *C. jejuni* (0.4%) isolates (Habib et al., 2009; Ellström et al., 2013; Ohishi et al., 2017), which might be due to the close phylogenetic relationship between *C. jejuni* isolates with LOS classes A and C (Gilbert et al., 2008). LOS group 2 classes (E, H, O, and P) are associated with ST-677 CC and ST-45 CC in *C. jejuni* bacteremia isolates (Ellström et al., 2013). However, another study found two other STs (ST-353 CC and ST-443 CC) for LOS group 2-related diarrheal *C. jejuni* strains in addition to ST-45 CC (Habib et al., 2009). Group 3 LOS class D have diarrheal *C. jejuni* strains (5%) that were assigned with ST-354 CC (Habib et al., 2009).

## Concluding Remarks

This review extends the *C. jejuni* LOS locus classification system. Currently, genomic-based classification of the LOS region is incomplete and vague. By providing a more refined classification system, investigators will be more readily able to link genomic class to LOS biosynthetic structures as they become available. Full LOS structural characterization is currently limited, so it is hard to determine whether locus classes will readily align with LOS structures and is clearly a focus for future research and may aid vaccine design. We also provide an overview of the frequency of *C. jejuni* LOS genotypes in *C. jejuni* populations originated from different sources and reviews the association between *C. jejuni* LOS locus genotypes and different human ganglioside-mimicking sialylated LOS structures. This review summarizes the various contributing factors in GBS development post-*Campylobacter* infection and shows that LOS group 1 containing LOS locus classes A, B, and C are commonly present in almost every type of *C. jejuni* population studied to date, regardless of its originating source.

## Author Contributions

AH wrote the first draft of the manuscript and prepared all figures. LM and AW made substantial and intellectual contributions to the work. All authors reviewed and/or edited the manuscript prior to submission and have approved the final version of manuscript as submitted.

## Conflict of Interest

The authors declare that the research was conducted in the absence of any commercial or financial relationships that could be construed as a potential conflict of interest.
